# Impacts of an Acute Care Telenursing Program on Discharge, Patient Experience, and Nursing Experience: Retrospective Cohort Comparison Study

**DOI:** 10.2196/54330

**Published:** 2024-04-04

**Authors:** Courtenay R Bruce, Steve Klahn, Lindsay Randle, Xin Li, Kelkar Sayali, Barbara Johnson, Melissa Gomez, Meagan Howard, Roberta Schwartz, Farzan Sasangohar

**Affiliations:** 1 Houston Methodist Houston, TX United States

**Keywords:** telenursing, telemedicine, patient discharge, health personnel, surveys and questionnaires, patient outcome assessment

## Abstract

**Background:**

Despite widespread growth of televisits and telemedicine, it is unclear how telenursing could be applied to augment nurse labor and support nursing.

**Objective:**

This study evaluated a large-scale acute care telenurse (ACTN) program to support web-based admission and discharge processes for hospitalized patients.

**Methods:**

A retrospective, observational cohort comparison was performed in a large academic hospital system (approximately 2100 beds) in Houston, Texas, comparing patients in our pilot units for the ACTN program (telenursing cohort) between June 15, 2022, and December 31, 2022, with patients who did not participate (nontelenursing cohort) in the same units and timeframe. We used a case mix index analysis to confirm comparable patient cases between groups. The outcomes investigated were patient experience, measured using the Hospital Consumer Assessment of Health Care Providers and Systems (HCAHCPS) survey; nursing experience, measured by a web-based questionnaire with quantitative multiple-choice and qualitative open-ended questions; time of discharge during the day (from electronic health record data); and duration of discharge education processes.

**Results:**

Case mix index analysis found no significant case differences between cohorts (*P*=.75). For the first 4 units that rolled out in phase 1, all units experienced improvement in at least 4 and up to 7 HCAHCPS domains. Scores for “communication with doctors” and “would recommend hospital” were improved significantly (*P*=.03 and *P*=.04, respectively) in 1 unit in phase 1. The impact of telenursing in phases 2 and 3 was mixed. However, “communication with doctors” was significantly improved in 2 units (*P*=.049 and *P*=.002), and the overall rating of the hospital and the ”would recommend hospital” scores were significantly improved in 1 unit (*P*=.02 and *P*=04, respectively). Of 289 nurses who were invited to participate in the survey, 106 completed the nursing experience survey (response rate 106/289, 36.7%). Of the 106 nurses, 101 (95.3%) indicated that the ACTN program was very helpful or somewhat helpful to them as bedside nurses. The only noticeable difference between the telenursing and nontelenursing cohorts for the time of day discharge was a shift in the volume of patients discharged before 2 PM compared to those discharged after 2 PM at a hospital-wide level. The ACTN admissions averaged 12 minutes and 6 seconds (SD 7 min and 29 s), and the discharges averaged 14 minutes and 51 seconds (SD 8 min and 10 s). The average duration for ACTN calls was 13 minutes and 17 seconds (SD 7 min and 52 s). Traditional cohort standard practice (nontelenursing cohort) of a bedside nurse engaging in discharge and admission processes was 45 minutes, consistent with our preimplementation time study.

**Conclusions:**

This study shows that ACTN programs are feasible and associated with improved outcomes for patient and nursing experience and reducing time allocated to admission and discharge education.

## Introduction

Telemedicine, particularly video televisits, has greatly expanded in the wake of the COVID-19 pandemic [1,2]. Televisits have shown promise as a robust, practical, efficacious, and scalable alternative to in-person office visits that could ameliorate labor supply shortages [3,4]. The published evidence suggests a generally positive attitude toward televisit appointments for chronic care, focused on addressing financial and transportation barriers and improving patients’ access to care [5-7]. Despite the promise shown by televisits, limited attention has been paid to applying this method in the acute care setting and, in particular, on how this promising technology can be leveraged to support nurses.

Estimates suggest that approximately 200,000 open nursing positions will become available each year between 2021 and 2031 [8]. Telenursing can augment nursing labor supply, decrease nursing workload, maintain patient and nurse safety, and positively impact nursing and patient experiences [9]. However, the impact of telenursing on outcomes in acute care settings remains a research gap.

To address this gap, this study aimed to evaluate the outcomes associated with a large-scale acute care telenurse (ACTN) program to support web-based admission and discharge processes for hospitalized patients compared to patients who did not undergo the ACTN program intervention. Admission and discharge are 2 substantive and time-consuming acute care nursing tasks that involve tedious documentation in the electronic health record (EHR) and extensive interaction with patients and families to gather history and provide patient education [10,11]. We aimed to develop an ACTN program to augment nursing care by conducting admission and discharge processes through telenursing in a large health system. Subsequently, we discuss the impacts on 4 end points: patient experience, nursing experience, time of discharge during the day, and length of time for discharge education processes. We hypothesized that the ACTN program would be associated with higher patient experience scores and improved nursing experience compared to standard admission and discharge practices.

## Methods

### Overview

This study was conducted in a large academic hospital system (approximately 2100 beds) in Houston, Texas. The preimplementation methods are reported more extensively in the studies by Hehman et al [12] and Schwartz et al [13]. Program implementation was first informed by nursing time and workload surveys and pilot implementation in 4 comparatively understaffed units. The chief innovation officer, along with nursing leaders and ACTN program administrators, met with the bedside nursing staff of these 4 understaffed units to solicit their input on where and how ACTN would add value to their workflow. Bedside nursing staff provided critical input on admission processes that could be delegated to individuals working remotely with no perceived negative impact on patient experience. We conducted participatory workflow design sessions with bedside nursing staff on the ACTN program to cocreate workflow integration points where the remote team could assist [13].

### Pilot Implementation and Procedures

Before implementation, the ACTN administrators trained bedside nurses in pilot units by demonstrating the use of technology during shift huddles. Then, the trainers presented slides on contact information and available support and provided a role demarcation process map, showing what the remote telenurse staff would be doing compared to what the bedside nurses needed to do to launch and conduct discharge education. Furthermore, the trainers invited the nursing staff to observe several discharges to learn how to conduct them. A software with Health Insurance Portability and Accountability Act compliance was uploaded to iPads (Apple Inc) and stored on each unit. Handheld iPads were available, and roaming iPads were made available for patients who could not hold an iPad.

**Figure 1:**
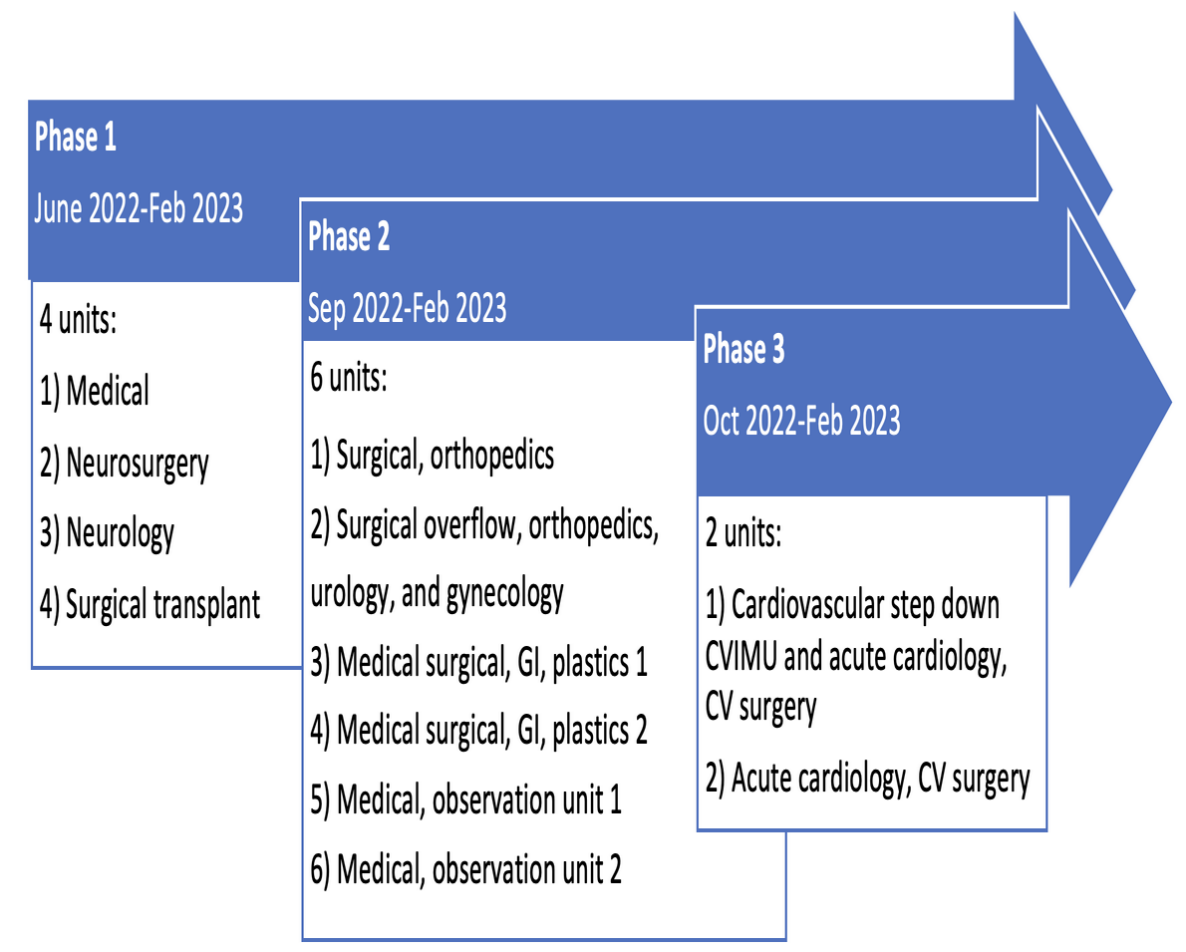
Overview of the 3-phase pilot implementation in 12 units. CV: cardiovascular; CVIMU: cardiovascular intermediate unit; GI: gastrointestinal.

The pilot implementation was staggered in a phased rollout, consisting of 3 sequenced phases, as shown in Figure 1. Upon admission, the acute care bedside nurse contextualized the ACTN program with patients and families by handing the patient an iPad with a preloaded remote program app (Caregility) and then pressing a soft key to allow the ACTN to enter the patient’s room via the iPad screen. The ACTN introduced themselves, completed the nursing admission profile in the EHR, placed a request for a consultation, and notified the bedside nurse that the admission was completed using secure SMS text messaging [13]. A similar process was followed for discharge workflow processes, where the ACTN completed patient education on discharge instructions, confirmed the patient’s pharmacy details, confirmed discharge transportation, and arranged for departure.

Bedside nurses used their discretion regarding which patients would be appropriate for the ACTN program. They based this determination principally on whether documentation was needed and whether the patient could benefit from the undivided attention the ACTN program could afford. Furthermore, they excluded patients from the ACTN program if the patients expressed discomfort using an iPad. After the initial rollout, patients’ input was sought on their experience with the ACTN program to identify where and how improvements could be made, and this feedback was incorporated into iterative revisions in subsequent rollouts.

### Pilot Outcomes Monitoring

A retrospective, observational cohort comparison was performed, in which all patients in our pilot units for the ACTN program (telenursing cohort) between June 15, 2022, and December 31, 2022, were compared with all patients who did not participate (nontelenursing cohort) in the same units in the same timeframe.

Our primary outcomes were patient experience and nursing experience. Patient experience scope was any process observable by patients [14]. We compared patient experiences in the telenursing and nontelenursing cohorts by evaluating patients’ responses to the widely used Hospital Consumer Assessment of Health Care Providers and Systems (HCAHPS) survey [15], which represented 8 aspects (called dimensions) of patient satisfaction. Each dimension was measured using a continuous variable (0 to 100 points).

For the telenursing cohort, we analyzed bedside nurses’ collective responses using a Forms (Microsoft Corp) survey conducted in April 2023. The survey consisted of 5 questions, asking them to indicate whether the ACTN program was helpful using a Likert scale with 5 items (very helpful to very unhelpful). Nurses were asked to provide open-ended comments to explain the reasons for their evaluation. At the end of the survey, we included 2 open-ended fields for nurses to describe opportunities for improvement in future rollouts and provide any additional comments.

Furthermore, we explored the time at which discharge occurred using the EHR admission, discharge, and transfer date and time. We compared the hour of the day the patient was discharged in the telenursing cohort with the hour of the day the patient was discharged in the nontelenursing cohort, hypothesizing a priori that patients might be discharged earlier in the day in the telenursing cohort. Finally, we analyzed the duration of discharge education for both cohorts, measured in minutes.

### Data Analysis

The patient demographic data were available for all patients. To confirm that the telenursing cohort had similar patient demographics as the nontelenursing cohort (and therefore to confirm that nurse biases in patient selection for the ACTN program were unlikely), we conducted a case mix index (CMI) evaluation. We first isolated the population of both cohorts into adults (aged ≥18 y). We compared only those patients who were discharged home and excluded those who were on extracorporeal membrane oxygenation or those who underwent a tracheostomy. The remaining population was evaluated to determine whether there was a difference in patient acuity and severity. After confirming that patient acuity and severity were of no significant difference, we included the inpatient and observation populations to evaluate the intervention results.

For the patient experience data, independent sample *t* tests (2-tailed) were used to compare the telenursing and nontelenursing cohorts across different HCAHPS dimensions and units. Analysis was conducted using R software (R Foundation for Statistical Computing). For the nursing experience survey data, we used Excel (Microsoft Corp) to analyze the responses to multiple-choice, discrete questions and thematic analysis to evaluate the open-text fields. Thematic analysis allows for eliciting key themes that emerge based on recurring statements [16]. The analysis followed an inductive approach. This approach uses open-ended questions, allowing themes to emerge with a few previously articulated assumptions on responses. Given the limited content, CRB served as the primary coder. Coding labels were used for data contextualizing, allowing for new themes to emerge throughout the coding process, using a codebook [16,17]. We stored emergent patterns and themes in an electronic format.

### Ethical Considerations

The hospital’s review board determined that the ACTN pilot would not be considered regulated human subjects research. All data reported in this study were aggregated and deidentified.

## Results

### Overview

The demographics of the telenursing and nontelenursing cohorts were relatively similar. Both cohorts had an average age of 60 years with an SD of 16.91; had a similar distribution in race and ethnicity (approximately 92/2319, 3.96% Asian; 525/2319, 22.64% Black; 425/2319, 18.33% Hispanic; 70/2319, 3.02% Native American, declined to identify, or other categories; and 1202/2319, 51.83% White); and had a similar distribution in female participants versus male participants (1249/2319, 53.86% vs 1070/2319, 46.14%). To further understand the population, the CMI analysis for acuity and severity showed that the CMI was slightly higher in the telenursing cohort than in the nontelenursing cohort, but the difference was not statistically significant (*P*=.75).

### Patient Experience

Among the first 4 units that rolled out in phase 1, all units experienced improvement in at least 4 and up to 7 HCAHPS domains (Table S1 in Multimedia Appendix 1). On average, 6 out of 8 HCAHPS domains were improved for patients in the telenursing cohort. All 4 units experienced improvements in the “overall rating” domain, and 3 of the 4 units experienced improvements in “likelihood to recommend” domain for patients in the telenursing cohort compared to those in the nontelenursing cohort within the same units. The improvement scores ranged from 1.4% for the neurosurgery unit (36 beds) to 11.6% for the medical unit (37 beds). Furthermore, all 4 units in the first phase of roll out experienced improved scores in the “responsiveness” domain by >4 points (ranging from 5% to 10.1%). A total of 2 out of the 4 units also experienced improvements in the “communication with nurses” (ranging from 1.7% to 3%) and “communication about medicines” (ranging from 3.3% to 11.7%) domains. The 2 units that did not experience improvement in the communication domains were the combined medical and surgery neurology and neurosurgical units (36 beds). Only the neurosurgical unit showed statistically significant improvements in 2 dimensions: “communication with doctors” (*P*=.03) and “would recommend hospital” (*P*=.04).

For the 7 units that rolled out during phase 2, only 1 orthopedic surgery unit (28 beds) experienced improvements in every domain (ranging from 0.9% to 12.5%). Medical observation unit 1 also improved in 5 areas. However, only improvements in “communication with doctors” (*P*=.002), “overall rating of hospital” (*P*=.02), and “would recommend hospital” (*P*=.04) were statistically significant*.* The remaining units experienced improvements in some domains for the telenursing cohort compared to the nontelenursing cohort, with no improvement in other domains. However, the scores for “communication with nurses” and “communication with doctors” domains were improved for most of the units that rolled out in phase 2 (Table S2 in Multimedia Appendix 1).

For the 2 units that rolled out in phase 3, both of which were surgical cardiac units with 36 beds, 1 unit experienced improvement in every domain except “responsiveness” (ranging from 1% to 12%). The other unit only experienced improvement in the “communication with doctors” (4.9%) and “care transitions” domains (1.1%). However, none of these improvements were statistically significant (Table S3 in Multimedia Appendix 1).

### Nursing Experience

Of the 289 nurses who were invited to participate in the survey, 106 completed the survey (36.7% response rate). Of the 106 nurses, 101 (95.3%) indicated that the ACTN program was “very helpful” or “somewhat helpful” to them as bedside nurses.

#### Quantitative Findings

The main reasons nurses gave for the program’s helpfulness included that it saved them time (94/106, 88.7%), allowed them to focus on more urgent clinical needs (90/106, 84.9%), allowed them to focus on activities they felt were more in line with their skill level (55/106, 51.9%), and allowed patients to have undivided attention for their discharge education (52/106, 49.1%). Among the 5 nurses who indicated that the ACTN program was somewhat unhelpful or very unhelpful, 3 (60%) indicated that workflows were not clear or needed further refinement or clarification. Furthermore, the nurse respondents shared several barriers and provided opportunities for improvement, with 91 (85.8%) out of 106 nurses offering suggestions.

#### Qualitative Findings

##### Overview

For the free-text explanation fields, all but 3 nurses (103/106, 97.2%) provided additional comments on the ACTN program helpfulness. Three themes emerged from the qualitative analysis of the free-text comments: (1) most of the nurses’ comments reflected that telenurses help bedside nurses save time, (2) respondents indicated that extra hands provided emotional and physical support in providing patient care, and (3) respondents perceived an improvement in patient safety by having a telenurse who could “catch missed” issues.

##### Time Saving

One of the perceived benefits of the telenursing program was saving time. One nurse said the following:

... Just putting in home medications alone takes up so much time. This new telenurse service helps

save time

Several nurses highlighted that admission and discharge processes are so complex and time-consuming that shifting this work to the ACTN program freed nurses to perform other activities, as reflected by this nurse:

The tele RN is able to spend as much time possible sufficiently educating an admission or discharge while allowing me time to respond to the needs of my other patients saving me time on one patient especially charting.

##### Emotional and Physical Support

For the second theme, several responses focused less on time management and perceived efficiencies and instead centered more on the emotional appeal and support in having an extra hand, as one nurse mentioned:

Being in such a fast-paced unit, it can be a bit stressful with so many discharges and admissions. Having a helpful hand is beneficial.

##### Improved Patient Safety

Finally, the third theme was perceived improvement in patient safety by having a telenurse who could “catch missed” issues (eg, an incorrectly identified pharmacy details), simultaneously allowing the primary bedside nurse to focus more intensely on other needs, essentially creating a 2-fold safety promotion. Some nurses noted that they could begin carrying out orders while the telenurses began completing the admission, facilitating quicker treatment and resolution of care needs, thereby improving the safety and quality of care. One nurse mentioned the following:

Allows [telenurses] to take on thorough and accurate admissions, while also preventing any rushing the patient might experience from the primary RN.

When asked for areas of improvement, the most recurring theme was having 24 hours of support during the weekend and during the week. The second theme for improvement was the reduced time to connect to a telenurse. The third theme was the availability of iPads. Nurses mentioned that iPads could sometimes be unavailable in patients’ rooms or they may not be fully charged.

### Time of Discharge

**Figure 2:**
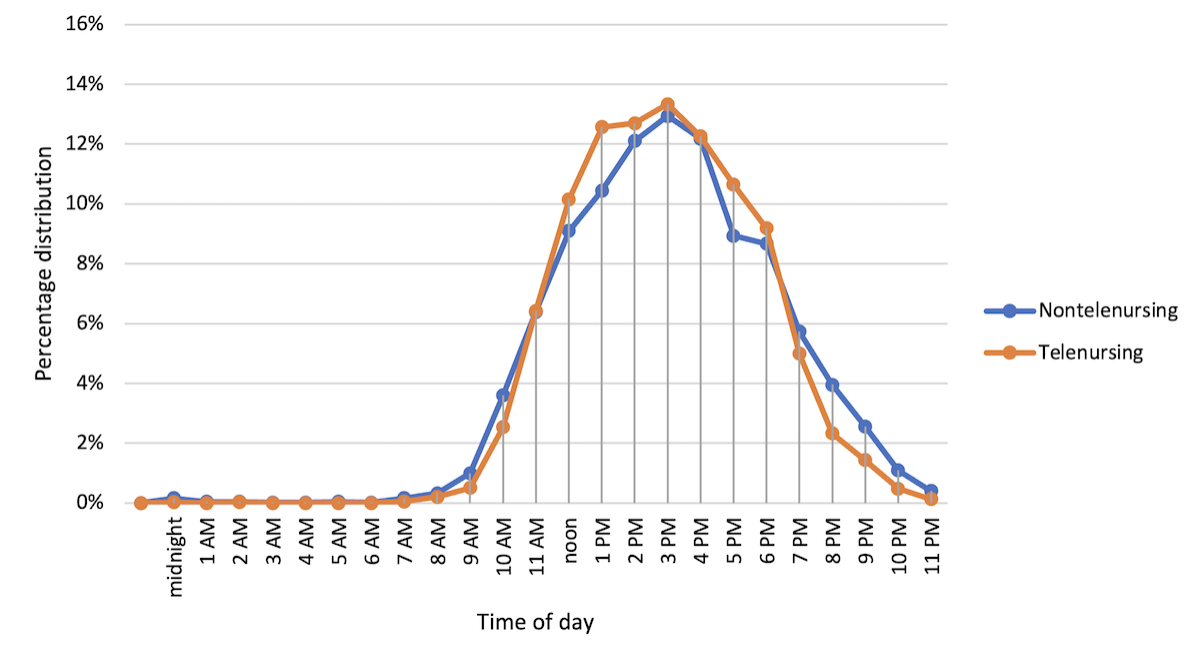
Comparison of percentage distribution of discharge by time of the day.

The time of day distribution is presented in Figure 2. The only noticeable difference between the telenursing and nontelenursing cohorts was a shift in the volume of patients discharged before 2 PM compared with those discharged after 2 PM at a hospital-wide level (Table 1). At an individual unit level, these results were not consistent and could be further explored by patient population and their needs to discharge. The variation was further illustrated when reviewing the length of stay of patients in the telenursing and nontelenursing cohorts. Only 5 out of the 12 units showed a decrease in the average inpatient length of stay.

**Table 1:** Time of the day distributions for the nontelenursing cohort compared to the telenursing cohort.

Hour of day	Nontelenursing (n=4220), n (%)	Telenursing (n=3907), n (%)
Before and up to 2 PM	1837 (43.53)	1766 (45.2)
After 2 PM	2383 (56.47)	2141 (54.8)

### Discharge Length

The ACTN admissions averaged 12 minutes and 6 seconds (SD 7 min and 29 s), and the discharges averaged 14 minutes and 51 seconds (SD 8 min and 10 s). The average duration for ACTN calls was 13 minutes and 17 seconds (SD 7 min and 52 s). Traditional cohort standard practice of a bedside nurse engaging in discharge and admission processes was 45 minutes, consistent with our preimplementation nursing time study.

## Discussion

### Principal Findings

Our results suggest that the ACTN program was associated with positive nursing experiences because it saved time. Furthermore, the ACTN program was associated with higher HCAHPS scores in several domains but only in the first series of units that piloted the intervention. In phase 1, the improvement in “communication with doctors” and “would recommend hospital” scores in 1 unit was statistically significant. In phase 2, the improvement in “communication with doctors” score was significant in 2 units and that in “overall rating of hospital” and “would recommend hospital” scores were significant in 1 unit. The time of day discharge was nearly the same in both the telenursing and nontelenursing cohorts. The duration for discharge processes was less than half in the ACTN cohort compared to the nonintervention cohort.

At the time of writing this paper, the United States was experiencing a critical nursing shortage that will likely reach an epidemic level in the next few decades [8]. Despite the promise shown by telenursing, to our knowledge, only 1 existing paper documents the impact of ACTN programs on HCAHPS-measured patient satisfaction using a small cohort of patients in a single, time-limited pre- and posttelenursing analysis [18]. A study by Schuelke et al [18] revealed a 6.2% increase in “communication with meds” and 12.7% increase in “communication with nursing” domain scores; other HCAHPS domains were not evaluated. This research builds upon the promising work of Schuelke et al [18], evaluating the impact of an ACTN program on several units with a much larger cohort of patients using a staggered rollout and comparing all HCAHPS domains between telenursing and nontelenursing cohorts within the same time frame and in the same units.

By conducting granular HCAHPS analyses, we identified what we believed to be a time sequence variability in that units that rolled out in phase 1 performed considerably stronger in HCAHPS impacts than units that rolled out in later phases. An explanation for this sequence effect might be that some later adopters had less potential for high effect size, given that the first 4 units of the rollout were specifically chosen for their staffing problems compared to later units. ACTN support might have augmented the staffing support to such a degree that allowed the impacts of the program to be more salient. An alternative explanation is that the early adopters and promoters tend to have greater diffusion uptake, greater saturation and adoptability, and greater impacts compared to late adopters or those resistant to adoption [19,20]. Our anecdotal evidence suggests that early adopters might have *wanted* the telenursing program to succeed; therefore, they applied consistent implementation practices to ensure success. Adopters in later stages were more aware of barriers and potential downsides and might have been more ambivalent about telenursing and, therefore, less likely to modify their behaviors to promote the telenursing program’s success.

Another interesting finding was that the ACTN program seemed to be effective for both medical and surgical units of all specialties. Phase 1 was a mix of medical and surgical units; however, all units experienced increases in scores. Phases 2 and 3 experienced mixed results, without a clear lead for one specialty over the other. This may suggest that ACTN programs are broadly applicable across acute settings and that success depends most crucially on the need and desire of unit leaders.

**Figure 3:**
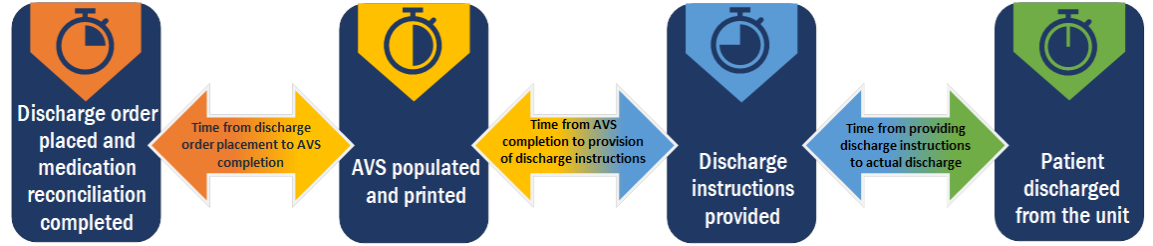
Overview of the discharge process in our health system. AVS: after-visit summary.

Our time of day discharge findings showed only a few quantitative positive efficiencies. However, our discharge duration analysis and nursing experience survey results showed that ACTN has major time-saving benefits for nurses, suggesting a discrepancy between perceived and actual time savings versus time-of-day discharge savings. One explanation for this discrepancy may be that many factors beyond nursing impact the time of the day a patient is discharged; therefore, while the bedside nurses’ time is saved, the remaining discharge processes beyond nurses remain unaffected. Specifically, there are 3 segments of time during discharge processes: (1) the time for the discharge order and medication reconciliation [21] to the time the after-visit summary (AVS) is populated and printed [22]; (2) the time the AVS is completed and printed to the time the discharge instructions are provided; and (3) the time from providing the discharge instructions to the actual discharge (Figure 3). Notably, telenurses’ involvement is currently limited to only the second segment of time. Specifically, telenurses’ involvement is not initiated until the AVS is printed by the nurse, which means that telenurses cannot positively impact any discharge activity that occurs between the time the discharge order is written and the time the AVS is printed. However, there are inefficiencies and bottlenecks in discharge processes that occur well before the AVS is printed [23,24]. For instance, the discharging physician may write a conditional discharge order early in the morning, listing conditions that cannot be fulfilled within a few hours or it may take bedside nursing longer than anticipated time to print the AVS.

To create a wider cascade effect for positively impacting the discharge processes for all segments of time, we are currently trying to obtain greater transparency through EHR reporting in what occurs for segments 1 and 3. For instance, at present, we know that at least 2 hospitals in our 8-hospital system have high incidence rates of conditional discharge orders that should be reduced. One hospital anecdotally reports that the discharging physician identifies incorrect pharmacies, which requires a nurse to send the scripts back to the discharging pharmacist to reconcile before discharge education can occur [25]; however, the prevalence and location of these issues remain speculative. Segment 3 is a black box of time [26]—the time it takes for hospital transport or an ambulance to arrive and move the patient to their destination and the time it takes for the family to pick up the patient. All these factors impact the discharge processes and need to be fully elucidated, explored, and streamlined. Furthermore, we hope to facilitate processes that enable telenurses to print the AVS, to remove the dependency on bedside nurses to begin the discharge education process.

### Limitations

This study has several noteworthy limitations. First, the study was conducted in 1 health system and the results may not be generalizable to other settings with different patient populations, processes, and implementation strategies [27]. Second, in this study, we did not control for other factors that could impact patient and provider satisfaction as well as discharge times; telenursing can only improve upon one component in a complex set of factors limiting discharge efficiency and satisfaction outcomes. Finally, participating nurses were aware of the ongoing study, and this knowledge might have affected their behavior [28].

### Future Directions

After the completion of this pilot study, the ACTN admission and discharge program has been rolled out to pilot medical units and all surgical and observation units. Our rationale for expansion rested on the premise that nursing experience is important to maintain and strengthen, particularly at a time when turnover is high in the health care industry in general. It is important to reduce staff inefficiencies in workload as a means of preserving or strengthening organizational morale and cost saving. Because our nursing experience findings for the ACTN program heavily supported the program, this served as the primary motivation for expansion. The nursing experience findings, coupled with the findings related to time-savings in discharge education and modest improvement, though not negative, in the HCAHPS findings for the ACTN program compared to the nontelenursing cohort, further supported expansion.

The initial scope for expansion included a complete system-wide implementation for all admissions and discharges. Furthermore, we are planning to expand the ACTN program beyond admissions and discharges. Responsive to qualitative feedback reported earlier, the next phase of the ACTN program will add safeguards on high-risk medications by having telenurses conduct double-checks, skin assessments, hourly rounding assistance, and auditing of safety functions and educational activities. These activities were chosen because they are time-intensive for nursing staff on the patient floors. Additional support in these areas would be a staff morale booster in addition to improved efficiencies for bedside nursing. Conducting hourly rounding using the ACTN program will require more time and resources; however, conducting high-quality, uninterrupted hourly rounds is known to be effective at improving patient safety and patient experience outcomes [29]. Therefore, we suspect that the ACTN program will have some positive impacts if rounds are consistently conducted, even if conducted virtually.

In addition, the ACTNs have been motivating other specialties to adopt or consider a similar program as the ACTN program to support stretched staffing. These specialties include respiratory care, in which virtual support can quickly identify patients in need of intensive on-site support; pharmacy, in which direct communication with staff on medications and patient training can happen through virtual means; infection control, in which room environments can be reviewed through virtual audits, moving quickly from floor to floor; and guest relations and spiritual care, in which patients can be visited virtually upon patient request. Furthermore, physicians who wish to either virtually enter inpatient rooms during their clinic days or from home can quickly drop in to see patients using the virtual program. For these groups to further develop advanced inpatient telemedicine programs, additional technology will be required, including cameras that can zoom into various portions of the room and advanced sound capabilities. Future work could expand programs similar to ACTN to specialties such as respiratory therapy, pharmacy, infection prevention, and spiritual care.

### Conclusions

This study provides preliminary evidence suggesting that telenursing may effectively address nursing shortages in acute care settings and positively impact patient and provider satisfaction as well as admission and discharge times. More work is needed to validate the findings in other settings, use other satisfaction metrics, and investigate the impact of telenursing on the quality of care and cost.

## Appendix

Multimedia Appendix 1 Additional outcome information for Hospital Consumer Assessment of Health Care Providers and Systems, time of day discharges, and discharge education processes.

## Abbreviations

ACTN: acute care telenurse
AVS: after-visit summary
CMI: case mix index
EHR: electronic health record
HCAHPS: Hospital Consumer Assessment of Health Care Providers and Systems

## Data Availability

Data sharing is not applicable as no data sets were generated during this study.
