# Supplementary material for: Impacts of an Acute Care Telenursing Program on Discharge, Patient Experience, and Nursing Experience: Retrospective Cohort Comparison Study
Source: J Med Internet Res. 2024 Apr 4;26:e54330. doi: 10.2196/54330 (PMC11027046; doi:10.2196/54330)
Supplement: Multimedia Appendix 1 [file jmir_v26i1e54330_app1.docx]

**Table 1.** Comparison of telenursing and nontelenursing HCAHPS^a^ scores in phase 1.

| Unit | Care transitions | | | | | *P* value | Communication about medicines | | | | | *P* value |
| --- | --- | --- | --- | --- | --- | --- | --- | --- | --- | --- | --- | --- |
|  | Telenursing | | Nontelenursing | | Score difference |  | Telenursing | | Nontelenursing | | Score difference |  |
|  | Score | Values, n | Score | Values, n |  |  | Score | Values, n | Score | Values, n |  |  |
| Medical | 43 | 151 | 54.1 | 211 | −11.1 | .136 | 49.1 | 110 | 37.4 | 148 | 11.7 | .501 |
| Neurosurgery. | *64.7* | 269 | *63.4* | 433 | 1.2 | .278 | *62.8* | 157 | *56.6* | 265 | 6.2 | .367 |
| Neurology | *55.9* | 183 | *50.4* | 299 | 5.6 | .756 | 55.7 | 105 | 59.9 | 175 | −4.2 | .815 |
| Surgical and transplant | 72 | 72 | 77 | 110 | −5 | .866 | 71.3 | 54 | 68 | 82 | 3.3 | .591 |
|  | Communication with doctors | | | | | *P* value | Communication with nurses | | | | | *P* value |
|  | Telenursing | | Nontelenursing | | Score difference |  | Telenursing | | Nontelenursing | | Score difference |  |
|  | Score | Values, n | Score | Values, n |  |  | Score | Values, n | Score | Values, n |  |  |
| Medical | *76* | 157 | *72.3* | 217 | 3.7 | .601 | *68.1* | 158 | *67.8* | 218 | 0.4 | .871 |
| Neurosurgery | *87.9* | 281 | *82.6* | 461 | 5.2 | .027 | *79.7* | 280 | *76.7* | 460 | 3 | .136 |
| Neurology | 83.4 | 182 | 87.1 | 306 | −3.6 | .883 | 76.6 | 184 | 79.8 | 308 | −3.2 | .439 |
| Surgical and Transplant | 83.3 | 74 | 92.6 | 112 | −9.3 | .088 | *84.2* | 74 | *82.5* | 112 | 1.7 | .064 |
|  | Discharge information | | | | | *P* value | Overall rating of hospital | | | | | *P* value |
|  | Telenursing | | Nontelenursing | | Score difference |  | Telenursing | | Nontelenursing | | Score difference |  |
|  | Score | Values, n | Score | Values, n |  |  | Score | Values, n | Score | Values, n |  |  |
| Medical | *81.3* | 150 | *75.2* | 198 | 6.1 | .618 | *71.9* | 153 | *60.3* | 213 | 11.6 | .330 |
| Neurosurgery | 89.3 | 263 | 92.7 | 359 | −3.3 | .914 | *82.7* | 272 | *81.2* | 442 | 1.5 | .056 |
| Neurology | 84.4 | 176 | 94.1 | 264 | −9.8 | .914 | *80.4* | 184 | *76.7* | 304 | 3.7 | .799 |
| Surgical and transplant | 94.9 | 68 | 88 | 92 | 6.9 | .733 | *89* | 73 | *84.4* | 111 | 4.6 | .438 |
|  | Responsiveness of hospital staff | | | | | *P* value | Would recommend hospital | | | | | *P* value |
|  | Telenursing | | Nontelenursing | | Score difference |  | Telenursing | | Nontelenursing | | Score difference |  |
|  | Score | Values, n | Score | Values, n |  |  | Score | Values, n | Score | Values, n |  |  |
| Medical | *53.7* | 149 | *43.6* | 209 | 10.1 | .909 | *71* | 155 | *65.7* | 213 | 5.3 | .308 |
| Neurosurgery | *64.5* | 264 | *55.3* | 436 | 9.2 | .232 | *86* | 272 | *84.5* | 440 | 1.4 | .043 |
| Neurology | *62.9* | 155 | *55.6* | 265 | 7.3 | .140 | *81.6* | 185 | *79.3* | 301 | 2.3 | .695 |
| Surgical and transplant | *77.4* | 73 | *72.4* | 109 | 5 | .385 | 91.8 | 73 | 94.6 | 111 | −2.8 | .089 |
|  |  |  |  |  |  |  |  |  |  |  |  |  |

^a^HCAHPS: Hospital Consumer Assessment of Health Care Providers and Systems.

**Table 2.** Comparison of telenursing and nontelenursing HCAHPS^a^ scores in phase 2.

| Unit | Care transitions | | | | | *P* value | Communication about meds | | | | | *P* value |
| --- | --- | --- | --- | --- | --- | --- | --- | --- | --- | --- | --- | --- |
|  | Telenursing | | Nontelenursing | | Score difference |  | Telenursing | | Nontelenursing | | Score difference |  |
|  | Score | Values, n | Score | Values, n |  |  | Score | Values, n | Score | Values, n |  |  |
| Surgery and orthopedics | 44.5 | 51 | 67.4 | 105 | −22.9 | .280 | 58 | 25 | 64 | 53 | −6 | .430 |
| SOOUGG^b^ | 53.8 | 79 | 51 | 147 | 2.8 | .294 | 51 | 52 | 50 | 100 | 0.9 | .456 |
| Medical-surgical GI^c^ and plastics 1 | 55.4 | 95 | 68.2 | 147 | −12.8 | .081 | 60.3 | 63 | 68.4 | 95 | −8 | .411 |
| Medical-surgical GI and plastics 2 | 61.2 | 100 | 63.1 | 166 | −1.9 | .785 | 54.2 | 59 | 64.6 | 99 | −10.4 | .514 |
| Medical observation 1 | *49.8* | 110 | *42.1* | 176 | 7.8 | .300 | *57.9* | 57 | *42.3* | 93 | 15.6 | .086 |
| Medical observation 2 | 52.5 | 53 | 65.7 | 75 | −13.2 | .066 | 56.3 | 24 | 80.2 | 36 | −24 | .659 |
|  | Communication with doctors | | | | | *P* value | Communication with nurses | | | | | *P* value |
|  | Telenursing | | Nontelenursing | | Score difference |  | Telenursing | | Nontelenursing | | Score difference |  |
|  | Score | Values, n | Score | Values, n |  |  | Score | Values, n | Score | Values, n |  |  |
| Surgery and orthopedics | 74.4 | 52 | 87.3 | 106 | −13 | .050 | 63.5 | 53 | 76.2 | 107 | −12.7 | .149 |
| SOOUGG^a^ | *82.9* | 84 | *76.6* | 152 | 6.3 | .563 | *75.9* | 83 | *74.5* | 151 | 1.4 | .632 |
| Medical-surgical GI and plastics 1 | *84* | 96 | *80.9* | 150 | 3.1 | .632 | *79.8* | 98 | *83.8* | 152 | −4.1 | .348 |
| Medical-surgical GI and plastics 2 | *90.3* | 106 | *82.5* | 174 | 7.7 | .208 | *78.4* | 105 | *77.2* | 175 | 1.2 | .782 |
| Medical observation 1 | *77.7* | 113 | *75.3* | 183 | 2.4 | .753 | *76.1* | 113 | *69.7* | 183 | 6.4 | .731 |
| Medical observation 2 | 75.3 | 58 | 80.2 | 82 | −4.9 | *.002* | 82.2 | 58 | 91.1 | 82 | −8.9 | .152 |
|  | Discharge information | | | | | *P* value | Overall rating of hospital | | | | | *P* value |
|  | Telenursing | | Nontelenursing | | Score difference |  | Telenursing | | Nontelenursing | | Score difference |  |
|  | Score | Values, n | Score | Values, n |  |  | Score | Values, n | Score | Values, n |  |  |
| Surgery and orthopedics | *86.9* | 42 | *86.1* | 78 | 0.8 | .750 | 66 | 50 | 84.7 | 104 | −18.7 | .129 |
| SOOUGG^a^ | *87* | 77 | *80.2* | 137 | 6.9 | .158 | *79.5* | 78 | *69* | 142 | 10.5 | .809 |
| Medical-surgical GI and plastics 1 | 88.3 | 90 | 90 | 140 | −1.6 | .833 | 83.2 | 95 | 84.6 | 147 | −1.4 | .300 |
| Medical-surgical GI and plastics 2 | *89.7* | 97 | *84.5* | 161 | 5.2 | .671 | 76.2 | 101 | 79.3 | 169 | −3.1 | .636 |
| Medical observation 1 | *76.2* | 105 | *73.4* | 165 | 2.8 | .403 | 69.7 | 109 | 71.8 | 173 | −2.1 | .924 |
| Medical observation 2 | 79.6 | 54 | 83.1 | 78 | −3.5 | .482 | 69.1 | 55 | 89.4 | 77 | −20.3 | *.024* |
|  | Responsiveness of hospital staff | | | | | *P* value | Would recommend hospital | | | | | *P* value |
|  | Telenursing | | Nontelenursing | | Score difference |  | Telenursing | | Nontelenursing | | Score difference |  |
|  | Score | Values, n | Score | Values, n |  |  | Score | Values, n | Score | Values, n |  |  |
| Surgery and orthopedics | 50 | 49 | 62.7 | 103 | −12.7 | .227 | 78.4 | 51 | 92.3 | 105 | −13.8 | .380 |
| SOOUGG^a^ | *69.5* | 77 | *60.8* | 143 | 8.7 | .984 | *83.3* | 78 | *70.8* | 146 | 12.5 | .329 |
| Medical-surgical GI and plastics 1 | 54.6 | 87 | 72.5 | 139 | −18 | .834 | *85.3* | 95 | *80.9* | 147 | 4.4 | .510 |
| Medical-surgical GI and plastics 2 | *62.1* | 91 | *58.6* | 161 | 3.4 | .518 | 75 | 100 | 79.3 | 168 | −4.3 | .730 |
| Medical observation 1 | 72.8 | 90 | 75.8 | 148 | −3 | .279 | 70.9 | 110 | 74.1 | 172 | −3.2 | .478 |
| Medical observation 2 | 64.1 | 46 | 74.3 | 70 | −10.2 | .480 | 75 | 52 | 89.8 | 74 | −14.8 | *.044* |

^a^HCAHPS: Hospital Consumer Assessment of Health Care Providers and Systems.

^b^SOOUGG: surgical-overflow and orthopedic, urology, gynecology, and gynecologic oncology.

^c^GI: gastrointestinal.

**Table 3.** Comparison of telenursing and nontelenursing HCAHPS^a^ scores for phase 3.

| Unit | Care transitions | | | | | *P* value | Communication about medicines | | | | | *P* value |
| --- | --- | --- | --- | --- | --- | --- | --- | --- | --- | --- | --- | --- |
|  | Telenursing | | Nontelenursing | | Score difference |  | Telenursing | | Nontelenursing | | Score difference |  |
|  | Score | Values, n | Score | Values, n |  |  | Score | Values, n | Score | Values, n |  |  |
| CV^b^ stepdown CVIMU^c^ and acute cardiology and CV surgery | 65.0 | 191 | 66.1 | 91 | 1.1 | .344 | *64.2* | 130 | *60.0* | 60 | −4.2 | .519 |
| Acute cardiology and CV surgery | 57.4 | 303 | 64.9 | 121 | 7.5 | .611 | 56.5 | 194 | 68.5 | 84 | 12.0 | .216 |
|  | Communication with doctors | | | | | *P* value | Communication with nurses | | | | | *P* value |
|  | Telenursing | | Nontelenursing | | Score difference |  | Telenursing | | Nontelenursing | | Score difference |  |
|  | Score | Values, n | Score | Values, n |  |  | Score | Values, n | Score | Values, n |  |  |
| CV stepdown CVIMU and acute cardiology and CV surgery | 85.3 | 196 | 90.2 | 92 | 4.9 | .428 | *81.4* | 195 | *78.8* | 91 | −2.6 | .074 |
| Acute cardiology and CV surgery | 85.7 | 309 | 87.5 | 123 | 1.9 | .450 | 76.3 | 308 | 83.3 | 124 | 7.0 | .506 |
|  | Discharge information | | | | | *P* value | Overall rating of hospital | | | | | *P* value |
|  | Telenursing | | Nontelenursing | | Score difference |  | Telenursing | | Nontelenursing | | Score difference |  |
|  | Score | Values, n | Score | Values, n |  |  | Score | Values, n | Score | Values, n |  |  |
| CV stepdown CVIMU and acute cardiology and CV surgery | *90.4* | 180 | *89.0* | 86 | −1.5 | .151 | *90.4* | 194 | *88.9* | 90 | −1.5 | .227 |
| Acute cardiology and CV surgery | 90.7 | 290 | 91.9 | 118 | 1.2 | .995 | 80.0 | 301 | 86.0 | 121 | 5.9 | .945 |
|  | Responsiveness of hospital staff | | | | | *P* value | Would recommend hospital | | | | | *P* value |
|  | Telenursing | | Nontelenursing | | Score difference |  | Telenursing | | Nontelenursing | | Score difference |  |
|  | Score | Values, n | Score | Values, n |  |  | Score | Values, n | Score | Values, n |  |  |
| CV stepdown CVIMU and acute cardiology and CV surgery | *67.7* | 169 | *60.8* | 79 | −6.9 | .850 | *92.3* | 195 | *91.2* | 91 | −1.1 | .841 |
| Acute cardiology and CV surgery | *68.1* | 266 | *59.0* | 106 | −9.1 | .427 | 81.0 | 298 | 89.2 | 120 | 8.2 | .590 |

^a^HCAHPS: Hospital Consumer Assessment of Health Care Providers and Systems.

^b^CV: cardiovascular.

^c^CVIMU: cardiovascular intermediate unit.
